# Comparisons of Esophageal Function Tests between Chinese and British Patients with Gastroesophageal Reflux Disease

**DOI:** 10.1155/2015/127275

**Published:** 2015-02-16

**Authors:** Feng Gao, Samantha Leach, Jian Yu Hao, Zhan Min Shang, Anthony Robert Hobson

**Affiliations:** ^1^Digestive Department, Beijing Chao-Yang Hospital, Capital Medical University, Beijing 100020, China; ^2^The Functional Gut Clinic, London W1G 6NB, UK

## Abstract

*Objective*. To investigate the esophageal function tests in British and Chinese patients with gastroesophageal reflux disease (GERD).* Methods*. Patients with GERD were selected from the functional gut clinic, London, and digestive department, Beijing Chao-Yang Hospital, after taking the examinations of High-resolution Manometry and Impedance (HRiM) and 24-hour Multi-Channel Intraluminal Impedance and pH Recording (MII/pH) between 2013 and 2014. Chinese healthy volunteers who undertook HRiM were also selected as control group.* Results*. Fifty-nine British and 82 Chinese patients with GERD and 62 Chinese healthy volunteers were entered. Values for British patients, Chinese patients, and healthy volunteers were as follows: Lower esophageal sphincter pressure (LESP) 16.0 ± 8.6, 16.5 ± 10.0, and 26.4 ± 10.9 mmHg, peristalsis (normal/small break/large break) 24/12/23, 44/10/28, and 57/1/4, total bolus transit time (TBTT) 7.3 ± 1.3, 7.6 ± 1.2, and 6.9 ± 0.9 s, and complete bolus transit rate (CBTR) 66.7 ± 37.8, 61.7 ± 36.4, and 90.3 ± 14.0%, respectively. Stepwise linear regression analysis showed that age, gender, and ethnicity did not have significant effect on LESP, TBTT, esophageal peristalsis, and CBTR in patients with GERD.* Conclusions*. British and Chinese patients with GERD presented similar values of LESP, TBTT, and impaired esophageal peristalsis and CBTR.

## 1. Introduction

The last ten years have been an exciting time in the study of esophageal motor disorders due to advances in esophageal function tests (EFT) methodology. Techniques, such as manometry, have enjoyed many improvements due to advances in transducer technology, computerization, and topographic colour-plot data presentation. In addition, the concomitant measurement of esophageal intraluminal impedance, which provides complementary data detailing functional bolus transit during manometry without the need for radiation, has increased the clinical utility of esophageal function test (EFT) [1–4]. At present high-resolution manometry and impedance (HRiM) and 24-hour multichannel intraluminal impedance and pH recording (MII/pH) are used to evaluate esophageal motor function, lower esophageal sphincter function, and pathophysiologic correlates of gastroesophageal reflux disease (GERD) and esophageal peristaltic performance [5, 6]. However, most of the studies were performed in western population; few were done in Chinese or oriental population. Therefore, we aimed to investigate the esophageal function among Chinese healthy volunteers and Chinese and British patients with GERD and explore the difference between British and Chinese patients with GERD.

## 2. Methods

### 2.1. Ethics

The examination of HRiM from Chinese healthy volunteers and patients received ethics approval from the Ethics Board of Beijing Chao Yang Hospital, Capital Medical University. And all British and Chinese participants gave written informed consent.

### 2.2. Patient Selection

Chinese healthy volunteers without symptoms and chronic disease undertook HRiM between January 2011 and June 2011. Chinese patients who presented GERD symptoms (mainly heartburn and regurgitation) with or with using proton pump inhibitors and undertook HRiM and MII/pH at the Digestive Department of Beijing Chao Yang Hospital between November 2013 and June 2014 were entered. British patients who presented GERD symptoms (mainly heartburn and regurgitation) with or with using proton pump inhibitors and undertook HRiM and MII/pH at the functional gut clinic of London between November 2013 and June 2014 were entered. Patients with other chronic active medical diseases (such as coronary artery disease, hypertension, malignancy, and diabetes mellitus) were excluded.

### 2.3. Stationary High-Resolution Esophageal Manometry and Impedance

A specially designed solid-state manometry catheter (Sandhill Scientific Inc., Highland Ranch, CO, USA) with 32 manometric sensors and four pairs of MII sensors separated by 5 cm interval was used to assess esophageal pressures and impedance with patient in the supine position. The lower esophageal sphincter (LES) was examined with the distal circumferential manometric sensors. The catheter was positioned so that the pressure transducers were located across the upper esophageal sphincter, esophageal body, LES and the distal channels were in the stomach. Ten swallows with 5 mL normal (0.9%) saline solution were then performed at 30-second intervals.

### 2.4. 24-Hour Oesophageal Multichannel Intraluminal Impedance and pH Recordings

The 2.1 mm outer diameter study catheter was comprised of six electrode pairs measuring intraluminal impedance of 3, 5, 7, 9, 15, and 17 cm above the LES, and an antimony pH sensor 5 cm above the LES (Sandhill Scientific Inc., Highland Ranch, CO, USA). An impedance amplifier delivered an ultra-low current in a range of 1-2 KHz with resulting current flow variations in response to intraluminal impedance changes (high impedance indicates gas or air; low impedance indicates liquid). The signals from six impedance channels and one pH channel are recorded at 50 samples per second. The data were stored in an ambulatory recorder and saved on a 256 MB CompactFlash card. Event markers recorded occurrence of symptoms, times of meals, and changes in body position. The study was performed as an outpatient after an overnight fast with LES located by esophageal manometry. The patients undertook HRiM and MII/pH with seven-day washout from proton pump inhibitors and/or H_2_ antagonists.

### 2.5. Data Collection

Esophageal bolus clearance can be assessed by measurement of total bolus transit time by classifying swallows as complete bolus transit (if bolus entry occurs at the most proximal site and bolus exit points are recorded in all three distal recording segments) or as incomplete bolus transit (if bolus exit is not identified at any of the three distal recording segments) and complete bolus transit rate. Distal contractile integral (DCI) of the distal segmental contraction is a parameter that integrates the length of the smooth muscle esophagus (cm), contractile pressure (mmHg), and durations (s) of contraction. Distal esophageal amplitude (DEA) is an average of contraction amplitude at 5 and 10 cm above the LES. Integrated relaxation pressure (IRP) reports mean EGJ pressure measured with an electronic equivalent of a sleeve sensor for four continuous or noncontinuous seconds of relaxation in the ten-second window following deglutitive UES relaxation. The parameters of length of lower esophageal sphincter (LESL), lower esophageal sphincter pressure (LESP), lower esophageal sphincter residual pressure (LESRP), and upper esophageal sphincter pressure (UESP) were also measured [1, 7]. The normal range for complete bolus clearance was complete liquid bolus transit rate (%) >79. The normal range for 20 mmHg isobaric contour breaks was 0–20% for >5 cm breaks (“large break”) and 0–30% for 2–5 cm breaks (“small break”) [1, 7, 8]. Patients were classified as weak peristalsis with small break if small break was 30–100%, weak peristalsis with large break if large break was 20–100%, and normal peristalsis if presenting normal range of peristaltic breaks. The parameters of Demeester score, acid exposure upright (%), acid exposure recumbent (%), acid exposure total (%), bolus exposure upright (%), bolus exposure recumbent (%), bolus exposure total (%), proximal acid episodes, proximal nonacid episodes, proximal total episodes, acid reflux episodes, nonacid reflux episodes, and total reflux episodes were measured [9]. All the values were measured by Bio View Analysis software (Sandhill Scientific, Inc., Highland Ranch, CO, USA).

### 2.6. Comparison Groups

There were three groups in the study, British patients with GERD, Chinese patients with GERD, and Chinese healthy volunteers. The diagnosis of GERD was according to the results of MII/pH, presenting an abnormal upright acid exposure time (≥6.3%) or recumbent acid exposure time (≥1.2%) or total acid exposure time (≥4.2%) [9].

### 2.7. Statistical Methods

Categorical data were described as the number and continuous data as mean ± SD. Data were analyzed using independent sampled *t*-test or chi-square test. Stepwise lineal regression analysis was performed to study the influence of independent variables on items of HRiM and MII/pH while controlling the effect of other variables, in which the dependent variables were items of HRiM and MII/pH; independents were age, gender, and ethnicity (Chinese and British) or group (Chinese healthy volunteers and GERD patients). A *P* value <0.05 was considered as statistically significant. All data were analyzed with SPSS 17.0.

## 3. Results

A total of 190 patients (88 British, 102 Chinese) who undertook HRM/Z and MII/pH with reflux symptoms were included in the study. 141 patients were diagnosed with GERD by MII/pH (59 British and 82 Chinese). 62 healthy volunteers were included in this study.

The results of HRiM and MII/pH between British patients and Chinese patients with GERD were shown in Table 1. Compared with Chinese patients, British patients had similar values for LESP, DEA, DCI, hiatus hernia rate, total bolus transit time, complete bolus transit rate, and peristalsis. Compared with Chinese patients, British patients presented significantly higher values of Demeester, acid exposure, bolus exposure, proximal reflux, and total reflux.

The results of the HRiM of Chinese healthy volunteers and patients with GERD were shown in Table 2. Compared with Chinese healthy volunteers, Chinese patients with GERD had significantly decreasing values for LESP, UESP, DEA, DCI, and complete bolus transit rate. Chinese patients with GERD presented significantly increasing values of hiatus hernia rate, total bolus transit time, and weak peristalsis.

The results of stepwise linear regression analysis of different demographic data on items of HRiM and MII/pH were shown in Table 3. The demographic data in the stepwise linear regression analysis explained 0.166 and 0.231 on IRP and UESP scores of HRiM as indicated by the *R* square. Taking UESP, for example, age and ethnicity had negative effect on UESP. Our model predicted decreasing score of UESP by 0.654 each increasing year of life and decreasing score of UESP by 27.506 in British patients against Chinese patients.

The results of stepwise linear regression analysis of different demographic data on items of HRiM and MII/pH were shown in Table 4. The demographic data in the stepwise linear regression analysis explained 0.431 on DCI scores of HRiM as indicated by the *R* square. Taking DCI, for example, age, gender, and group had negative effect on DCI. Our model predicted decreasing score of DCI by 13.082 each increasing year of life, decreasing score of DCI by 438.073 in female, and decreasing score of DCI by 1668.335 in Chinese GERD patients.

## 4. Discussion

This study provides a set of esophageal HRiM and MII/pH values obtained in London center and Beijing center in patients with GERD and healthy volunteers. All measurements were performed with the Sandhill system, which is a solid-state HRiM and MII/pH assembly.

At present, most of the studies on EFT and GERD were done on the western population; few were done on Chinese or oriental population. In this study, we compared esophageal HRiM and MII/Z values between British and Chinese patients with GERD with the same Sandhill system and in the supine position. British patients presented younger age, longer length of LES, and lower LESRP, IRP, and UESP. MII/pH showed British patients presented more acid exposure, bolus exposure, and reflux episodes than Chinese patients. The difference may be oriented from different lifestyle and racial background of western and Chinese populations.

Our study evaluated the factors contributing to values of HRiM in patients with GERD, such as age, gender, and ethnicity (British and Chinese). Stepwise linear regression analysis showed that age, gender, and ethnicity did not have significant effect on LESP, total bolus transit time, esophageal function of peristalsis, and bolus clearance in patients with GERD. Our study also evaluated the factors contributing to values of HRiM in Chinese, such as age, gender, and group (healthy volunteers and patients with GERD). Stepwise linear regression analysis showed that present GERD had significantly negative effect on LESP, LESRP, UESP, DEA, DCI, and CBTR and positive effect on poor peristalsis and TBTT. Female gender had significantly positive effect on poor peristalsis.

The primary determinants of GERD severity are a dysfunctional antireflux barrier and impaired esophageal clearance. The antireflux prevents reflux of gastric contents into the esophagus, while peristalsis helps to clear the reflux in order to reduce exposure to the noxious components of the gastric juice. The primary mechanisms of reflux have focused on three dominant mechanisms: (1) transient LES relaxation, without anatomic abnormality, (2) LES hypotension, again without anatomic abnormality, or (3) anatomic distortion of the EGJ inclusive of (but not limited to) hiatus hernia. Once the gastroesophageal reflux enters the esophagus, peristalsis functions to clear the esophagus of the refluxate. Defects in the integrity of the peristaltic wave will lead to impaired bolus transit and prolonged esophageal acid exposure [6]. Bulsiewicz et al. [10] reported that longer breaks in the peristaltic wave predicted incomplete bolus clearance. Ribolsi et al. [11] reported that weak peristalsis with large break was associated with high acid exposure and delayed reflux clearance in the supine position in GERD patients. In our study, we found British and Chinese GERD patients presented similar increasing values of hiatus hernia rate, total bolus transit time, and weak peristalsis and decreasing values of LESP and complete bolus clearance rate, which confirmed the primary mechanisms of LES hypotension, anatomic distortion of the EGJ, and impaired peristalsis.

Studies performed in western countries have already explored the normal range for MII/pH monitoring [9]. However, the diversity in dietary habit, life style, and body build between western and eastern populations has led us to hypothesize that Chinese might have a different normal range for reflux episodes. In a southern Chinese study [12], the number of reflux episodes in the Chinese population was similar to that in the western population, and the normal values of acid exposure time in the Chinese population were lower than those of western population. But these normal values may not apply to the whole Chinese population and we use the western normal range in this study.

Our study has some limitations. All subjects were recruited from two centers in two cities using the western normal range for MII/pH, and lack of symptoms severity score might have potential selection bias. Absence of British healthy control, Chinese healthy volunteers' age significantly younger comparing to Chinese GERD patients, and the small number of patients limited the statistical power of the study. However, we were the first to compare western and oriental population with the same examining system.

In summary, British and Chinese patients with GERD presented similar values of LESP, DCI, hiatus hernia rate, total bolus transit time, and impaired esophageal function of peristalsis and bolus clearance.

## Figures and Tables

**Table 1:** Demographic data and high-resolution manometry and impedance results and 24-hour multichannel intraluminal impedance and pH recording of different groups.

Items	British	Chinese	Independent-sample *t*-test or chi-square
	*n* = 59	*n* = 82	
Age (mean ± SD, yr)	48.8 ± 13.4	53.5 ± 11.8	*P* = 0.027
Male/Female, *n*	28/31	39/43	*P* = 0.990
BMI	24.6 ± 1.9	25.1 ± 2.1	*P* = 0.136
LESP (mean ± SD, mmHg)	16.0 ± 8.6	16.5 ± 10.0	*P* = 0.758
LESL (mean ± SD, cm)	3.1 ± 0.3	2.9 ± 0.5	*P* = 0.001
LESRP (mean ± SD, mmHg)	2.6 ± 5.0	4.4 ± 4.7	*P* = 0.029
IRP (mean ± SD, mmHg)	4.1 ± 4.6	9.0 ± 5.9	*P < 0.001 *
UESP (mean ± SD, mmHg)	50.0 ± 30.8	75.0 ± 25.6	*P < 0.001 *
DEA (mean ± SD, mmHg)	55.7 ± 30.2	58.9 ± 31.9	*P* = 0.554
DCI (mean ± SD, mmHg·cm·s)	533.0 ± 540.1	535.8 ± 556.6	*P* = 0.976
Peristalsis *n* normal/small break/large break	24/12/23	44/10/28	*P* = 0.237
Total bolus transit time (s)	7.3 ± 1.3	7.6 ± 1.2	*P* = 0.229
Complete bolus transit rate (%)	66.7 ± 37.8	61.7 ± 36.4	*P* = 0.471
Hiatus hernia *n* (%)	6 (10.1)	9 (10.9)	*P* = 0.878
Demeester	24.4 ± 24.2	15.0 ± 12.8	*P* = 0.008
Acid exposure upright (%)	7.6 ± 7.8	5.0 ± 5.5	*P* = 0.028
Acid exposure recumbent (%)	6.6 ± 11.1	3.3 ± 5.0	*P* = 0.035
Acid exposure total (%)	6.6 ± 7.0	4.1 ± 4.2	*P* = 0.018
Bolus exposure upright (%)	5.4 ± 4.6	3.7 ± 0.5	*P* = 0.019
Bolus exposure recumbent (%)	1.6 ± 3.2	1.2 ± 2.1	*P* = 0.374
Bolus exposure total (%)	3.7 ± 3.6	2.5 ± 2.1	*P* = 0.023
Proximal acid (*n*)	30.3 ± 19.2	13.0 ± 9.4	*P < 0.001 *
Proximal nonacid (*n*)	17.2 ± 15.1	14.5 ± 12.7	*P* = 0.284
Proximal total (*n*)	47.2 ± 23.5	27.4 ± 16.1	*P < 0.001 *
Acid reflux (*n*)	40.1 ± 23.1	20.9 ± 12.8	*P < 0.001 *
Nonacid reflux (*n*)	34.0 ± 34.7	22.7 ± 15.8	*P* = 0.023
Total reflux (*n*)	74.8 ± 42.8	43.6 ± 24.0	*P < 0.001 *

BMI: body mass index; LESP: lower esophageal sphincter pressure; LESL: length of lower esophageal sphincter; LESRP: lower esophageal sphincter residual pressure; IRP: integrated relaxation pressure; UESP: upper esophageal sphincter pressure; DEA: distal esophageal amplitude; DCI distal contractile integral.

**Table 2:** Demographic data and high-resolution manometry and impedance results of different Chinese groups.

Items	Healthy volunteers	GERD	Independent-sample *t*-test or chi-square
	*n* = 62	*n* = 82	
Age (mean ± SD, yr)	32.0 ± 11.2	53.5 ± 11.8	*P < 0.001 *
Male/female, *n*	25/37	39/43	*P* = 0.387
LESP (mean ± SD, mmHg)	26.4 ± 10.9	16.5 ± 10.0	*P < 0.001 *
LESL (mean ± SD, cm)	2.7 ± 0.4	2.9 ± 0.5	*P* = 0.135
LESRP (mean ± SD, mmHg)	6.6 ± 4.7	4.4 ± 4.7	*P* = 0.005
IRP (mean ± SD, mmHg)	10.4 ± 4.9	9.0 ± 5.9	*P = 0.142 *
UESP (mean ± SD, mmHg)	101.4 ± 49.5	75.0 ± 25.6	*P < 0.001 *
DEA (mean ± SD, mmHg)	95.3 ± 35.4	58.9 ± 31.9	*P < 0.001 *
DCI (mean ± SD, mmHg·cm·s)	1891.5 ± 1131.9	535.8 ± 556.6	*P < 0.001 *
Peristalsis *n* Normal/small break/large break	57/1/4	44/10/28	*P < 0.001 *
Total bolus transit time (s)	6.9 ± 0.9	7.6 ± 1.2	*P* = 0.001
Complete bolus transit rate (%)	90.3 ± 14.0	61.7 ± 36.4	*P < 0.001 *
Hiatus hernia *n* (%)	1 (1.6)	9 (10.9)	*P* = 0.029

LESP: lower esophageal sphincter pressure; LESL: length of lower esophageal sphincter; LESRP: lower esophageal sphincter residual pressure; IRP: integrated relaxation pressure; UESP: upper esophageal sphincter pressure; DEA: distal esophageal amplitude; DCI distal contractile integral.

**Table 3:** Results of stepwise linear regression analysis of different demographic data on items of HRiM and MII/pH in British and Chinese GERD patients.

HRiM and MII/pH items	Unstandardized coefficients (*n* = 141)				
	Constant	Age	Gender	Ethnicity	*R* square
LESP	—	—	—	—	—
LESL	2.428	—	—	0.266	0.071
LESRP	—	—	—	—	—
IRP	16.775	—	—	−4.374	0.166
UESP	124.142	−0.654	—	−27.566	0.231
DEA	—	—	—	—	—
DCI	—	—	—	—	—
Peristalsis	—	—	—	—	—
TBTT	—	—	—	—	—
CRTR	—	—	—	—	—
Demeester	−10.667	0.286	—	10.366	0.090
Acid exposure upright	−4.652	0.126	—	2.989	0.086
Acid exposure recumbent	0.037	0.058	—	3.244	0.037
Acid exposure total	−3.099	0.084	—	2.762	0.077
Bolus exposure upright	2.197	—	—	1.593	0.042
Bolus exposure recumbent	—	—	—	—	—
Bolus exposure total	1.323	—	—	1.192	0.041
Proximal acid	9.415	−0.228	—	15.800	0.278
Proximal nonacid	23.882	—	−5.456	—	0.039
Proximal total	11.390	—	−9.528	24.536	0.349
Acid reflux	16.076	—	−8.050	17.171	0.244
Nonacid reflux	11.006	—	—	11.714	0.050
Total reflux	32.592	—	−11.691	28.912	0.206

LESP: lower esophageal sphincter pressure; LESL: length of lower esophageal sphincter; LESRP: lower esophageal sphincter residual pressure; IRP: integrated relaxation pressure; UESP: upper esophageal sphincter pressure; DEA: distal esophageal amplitude; DCI distal contractile integral; TBTT: total bolus transit time; CBTR: complete bolus transit rate. Only data with *P* < 0.05 were expressed as values of beta-coefficients. “—”: *P* > 0.05.

**Table 4:** Results of stepwise linear regression analysis of different demographic data on items of HRiM in Chinese healthy volunteers and GERD patients.

HRiM items	Unstandardized coefficients (*n* = 144)				
	Constant	Age	Gender	Group	*R* square
LESP	36.213	—	—	−9.824	0.181
LESL	—	—	—	—	—
LESRP	8.962	—	—	−2.275	0.054
IRP	—	—	—	—	—
UESP	130.787	—	—	−1.002	0.157
DEA	131.731	—	—	−36.408	0.227
DCI	3840.796	−13.082	−438.073	−1668.335	0.431
Peristalsis	0.060	—	0.255	0.678	0.177
TBTT	6.694	—	—	0.662	0.080
CRTR	93.454	—	—	−15.873	0.063

LESP: lower esophageal sphincter pressure; LESL: length of lower esophageal sphincter; LESRP: lower esophageal sphincter residual pressure; IRP: integrated relaxation pressure; UESP: upper esophageal sphincter pressure; DEA: distal esophageal amplitude; DCI distal contractile integral; TBTT: total bolus transit time; CBTR: complete bolus transit rate. Only data with *P* < 0.05 were expressed as values of beta-coefficients. “—”: *P* > 0.05.
